# Importance of Clinical Isolates in *Cryptococcus neoformans* Research

**DOI:** 10.3390/jof9030364

**Published:** 2023-03-16

**Authors:** Katrina M. Jackson, Minna Ding, Kirsten Nielsen

**Affiliations:** Department of Microbiology and Immunology, University of Minnesota, Minneapolis, MN 55455, USA

**Keywords:** *Cryptococcus neoformans*, clinical isolates, host–pathogen interaction, genotype

## Abstract

The human pathogenic fungus *Cryptococcus neoformans* is a global health concern. Previous research in the field has focused on studies using reference strains to identify virulence factors, generate mutant libraries, define genomic structures, and perform functional studies. In this review, we discuss the benefits and drawbacks of using reference strains to study *C. neoformans*, describe how the study of clinical isolates has expanded our understanding of pathogenesis, and highlight how studies using clinical isolates can further develop our understanding of the host–pathogen interaction during *C. neoformans* infection.

## 1. Introduction

Almost all cases of human cryptococcal disease are caused by either the *Cryptococcus neoformans* or *Cryptococcus gattii* species complexes [1]. The *C. neoformans* complex causes the majority of human disease and contains the species *C. neoformans* and *C. deneoformans* [1]. *C. neoformans* is widely distributed in the environment, is found on all continents except for Antarctica, and causes the vast majority of human disease. In contrast, human disease due to *C. deneoformans* is most frequently observed in Europe, where it can account for up to 20% of human disease. *C. gattii* infections are rare but are seen in both immunocompromised and apparently healthy individuals. Disease due to *C. neoformans* and *C. deneoformans* is predominantly seen in severely immunocompromised individuals, such as cancer chemotherapy and organ transplant patients, as well as individuals with advanced HIV/AIDS. Due to the HIV pandemic and increased reliance on immunosuppressive treatments, cryptococcosis—specifically, cryptococcal meningitis caused by *C. neoformans*—transitioned from a relatively rare disease predominantly observed in pigeon farriers to a disease of global significance.

*C. neoformans* is a leading cause of HIV/AIDS-related mortality, with an estimated global incidence rate of 223,100 per year [2]. In countries with limited access to medical resources, cryptococcal meningitis in individuals with advanced HIV/AIDS has a mortality rate of up to 40% [3]. Even in well-resourced settings, treatment for HIV-associated cryptococcal meningitis remains challenging, with the mortality rate still above 20% [4]. In individuals with HIV and cryptococcal meningitis, the timing of antiretroviral therapy is critical for reducing the risk of immune reconstitution inflammatory syndrome (IRIS)—a paradoxical reaction that occurs during immunological recovery with antiretroviral therapy (ART) despite effective cryptococcal meningitis therapy. Deferring ART until after the diagnosis and treatment of cryptococcal meningitis significantly improves patient survival [5]. Unfortunately, recommended treatments for cryptococcal meningitis frequently involve antifungal drugs that require costly laboratory monitoring and have associated toxicities with inconsistent treatment protocols [6,7]. The addition of therapeutic lumbar punctures to reduce intracranial pressure, intravenous fluid hydration, and electrolyte supplementation further complicate cryptococcal meningitis treatment and likely impact patient survival outcomes [3].

*C. neoformans* is genetically divided into three major clades: VNI, VNII, and VNB. VNI and VNII are both predominantly clonal and distributed globally [8,9], while VNB is highly diverse and predominantly seen in sub-Saharan Africa and South America [10]. The vast majority of clinical isolates studied to date are from the VNI lineage, including the H99 reference strain and the various H99 derivatives, such as the commonly used reference strain KN99α [11,12]. While H99 is a VNI clinical isolate, it came from a Hodgkin’s lymphoma patient and is also not very closely related to most clinical isolates collected from patients with HIV [13]. Thus, whether the H99 isolate is representative of the majority of *C. neoformans* infections has been questioned.

## 2. Advantages and Disadvantages of Reference Isolates

**Figure 1:**
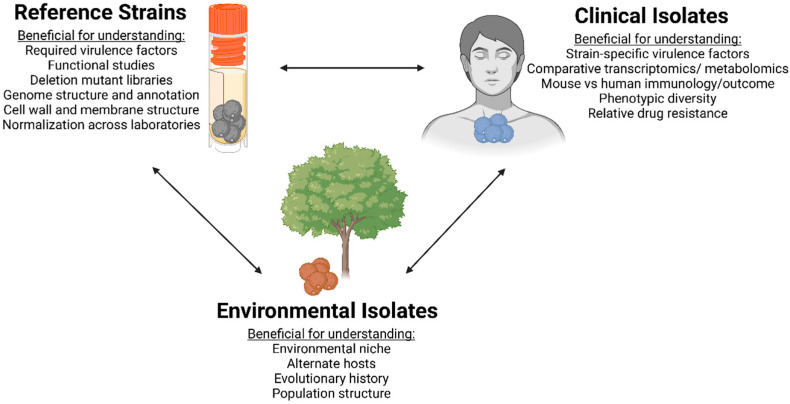
References strains, clinical isolates, and environmental isolates are beneficial for studying various aspects of Cryptococcus biology.

Much research to date on the *Cryptococcus* species complex comes from studies of reference isolates (Figure 1). The first reference isolates that were widely adopted by the research community were the *C. deneoformans* congenic strains JEC20 and JEC21, and the 52D strain often used for immunological studies. The JEC20/JEC21 strains were derived from a cross between the environmental isolate NIH433 and the clinical isolate NIH12, followed by backcrossing into an **a** mating-type progeny from this cross [12,14]. However, since epidemiological studies revealed that most clinical isolates were *C. neoformans* [15,16], many researchers shifted to the *C. neoformans* clinical isolate H99 as a reference strain. H99 was isolated at Duke University from a Hodgkin’s lymphoma patient in 1978 [17]. Genomic studies later revealed that genetic variation occurred as H99 was passed between various laboratories, resulting in a cluster of related strains with variable phenotypes and genotypes [18,19]. These variations include the original H99O strain, a less virulent H99W (wimp) isolate, and H99E (eunuch)—a strain that does not mate [18,19]. Additionally, H99 was passaged through a rabbit model, generating the highly virulent H99S (stud) strain [18,19]. To overcome the use of multiple variants of H99 across laboratories, the congenic strains KN99**a** and KN99α were developed in the H99S genetic background in 2003 [12], distributed throughout the research community, and are now the most used reference strains by the research community.

The use of reference strains in *C. neoformans* has resulted in major advances in our understanding of the biology and pathogenesis of this important pathogen. Studies have used the H99/KN99 strains to generate an annotated genome [17], make a gene deletion library for most *C. neoformans* genes [20,21], decipher the transcriptome [22,23,24,25,26,27] and metabolome [28,29], characterize the cell wall and membrane [30,31], and analyze host–pathogen interactions in mice [32,33]. The depth of studies using reference strains is beneficial for researchers interested in detailed functional analyses, and the deletion mutant library in KN99α promotes rapid characterization of genes with undefined functions in *C. neoformans*.

However, H99 and KN99α represent only a single strain’s genetic background, and recent population genetic and epidemiological studies show that H99 is evolutionarily distinct from many of the isolates commonly identified in patients with cryptococcosis [1,13]. Thus, while H99 is likely an excellent reference for understanding the basic biology of *C. neoformans*, we should use caution when H99 is used for studies focused on the pathogenesis and clinical outcomes of disease. For example, consider the applicability of reference strains in the study of latent *C. neoformans* infections. *C. neoformans* is known to cause a latent, subclinical infection in 99% of the human population, although some researchers suggest that it could be considered to be a member of the lung microbiome [34]. Cryptococcosis is thought be caused by the reactivation of latent infection in the context of immune deficiency [35,36] However, H99 is highly pathogenic in animal models of cryptococcosis, and even as few as five cells can cause lethal disease [37], precluding its use for latent infection models. Instead, another reference strain, 52D, was initially used to develop latent models of cryptococcosis [38,39,40]. However, 52D is a *C. deneoformans* reference strain, and this species only causes 4% of infections in cryptococcal meningitis patients overall and only 1% in patients with HIV, as opposed to *C. neoformans*, which causes 95% and <99% of cryptococcosis cases, respectively [1]. To overcome this issue, we turned to clinical isolates to identify *C. neoformans* strains capable of establishing latent infections and used these strains to develop a latent murine model of cryptococcosis [35].

Importantly, the use of reference strains also does not take into account the variation in patient disease presentation and outcomes observed in cryptococcosis [41,42,43,44]. The use of H99/KN99α as reference strains in pathogenesis studies does not capture the breadth of phenotypic and clinical variations that are found in natural populations of *C. neoformans* clinical isolates. Thus, to gain a more complete understanding of the clinical course of cryptococcosis, we need to extend our research beyond reference isolates and take advantage of the diversity of clinical phenotypes associated with disease outcomes in humans.

## 3. Clinical and Phenotypic Variation in Clinical Isolates

Clinical isolates are a valuable resource for researchers who want to perform translational studies that impact patient clinical outcomes (Figure 1). Cryptococcosis—specifically, cryptococcal meningitis—has highly variable disease presentation and outcomes, with some of this variability linked to differences in the immune responses of patients [45,46,47,48,49,50]. Intriguingly, a recent study by Mukaremera and colleagues showed that *C. neoformans* clinical isolates obtained from individuals with HIV exhibited equivalent survival outcomes in a murine model of cryptococcosis [51]. Isolates from patients who rapidly succumbed to the infection proved to be highly virulent, with rapid mortality in the murine model. Similarly, mice infected with strains from patients who survived the infection tended to produce attenuated infections and longer survival in the murine model [51]. Importantly, this study also identified latent infections in mice and laid the foundation for developing a murine model for latent infection and reactivation following immune deficiency [35].

In contrast, studies with clinical isolates have also identified strains that behave differently in murine models compared to humans. For example, sequence type 5 (ST5) isolates predominantly cause disease in immunocompetent individuals in Asia [13,44,52,53]. Paradoxically, animal models comparing ST5 and non-ST5 clinical isolates revealed that non-ST5 isolates were more lethal in mice, contrary to the human outcomes [54]. Surprisingly, the non-ST5 clinical isolates had a significant increase in the cytokine TNFα in the murine model [54], even though this cytokine was associated with improved survival in humans [55,56]. Better understanding of why some clinical isolates produce equivalent disease outcomes in humans and mice, whereas other isolates produce opposite outcomes, will be critical to our interpretation and identification of important host–pathogen interactions that alter the course of human disease.

Studies with clinical isolates can also highlight the major gaps in our understanding of the many interactions between *C. neoformans* and the host’s immune system, and how these interactions lead to differences in patient disease outcomes. For example, early studies with H99/KN99α reference strains suggested a binary interaction in which type-1 immune responses are protective and type-2 responses are detrimental [57,58,59]. However, as more studies are performed with clinical isolates, it has become increasingly apparent that the host–pathogen interaction is not as clear-cut as this binary model proposes. Instead, the parabolic damage–response model proposed by Pirofski and Casadevall appears to be more accurate, with too little immune response resulting in disease, but an overactive immune response can also enhance disease [60,61]. Because *C. neoformans* is not an obligate human pathogen, it is quite likely that convergent evolution occurred across various global lineages. Development of treatment strategies that target the host–pathogen interaction will only be successful if we identify the similarities and differences in the host–pathogen interactions across various lineages.

Studies using clinical isolates are also beneficial when identifying *C. neoformans* traits that are critical for virulence (Figure 1). Infections by *C. neoformans* are acquired from the environment, and most environmental isolates have attenuated virulence compared to genetically similar clinical isolates [62,63]. Differences in virulence are likely to be a complex balance between the arsenal of virulence factors that a strain possesses and the arsenal of defenses that the host has at their disposal [60,61,64,65]. Studies with reference strains have identified the basic virulence factors that all *C. neoformans* strains must possess to cause disease—essentially, the factors that allow *C. neoformans* to be a pathogen—such as capsule formation, melanin production, and the ability to grow at 37 °C [66,67]. However, there are many other factors in *C. neoformans* that either enhance or reduce virulence in ways that we do not yet fully understand. Strains isolated from the environment that are within the same sequence type and, thus, very closely related to clinical isolates often have reduced virulence in murine models of cryptococcosis [63,68,69]. While the differences between closely related environmental and clinical isolates that affect virulence are unknown, it is hypothesized that the clinical isolates will contain changes in genes that promote virulence. Desjardins et al. performed a genome-wide association study (GWAS) searching for small polymorphisms and loss-of-function mutations that separated clinical isolates from environmental isolates. They found that most of the genetic changes occurred in genes involved in the oxidative stress response, filamentation, membrane and cell wall integrity, and responses to nitrosative stress [70]. In a follow-up study, the authors analyzed the transcriptomes of clinical and environmental isolates when grown in various in vitro conditions and compared them to transcriptional profiles in rabbit cerebrospinal fluid (CSF) [71]. While this study showed that differentially expressed genes were associated with genotype (i.e., isolates that were closely related had similar transcriptomes), it was still able to identify a core group of genes that were upregulated in rabbit CSF across all the isolates, suggesting that these genes are likely critical for the ability of *C. neoformans* to survive in the host [71]. Interestingly, the authors identified only minor transcriptional differences between the clinical and environmental isolates [62], indicating that the observed differences in virulence may be due to small polymorphisms in a select subset of genes—most likely conferred by in vivo selection. Consistent with these data, Gerstein and colleagues identified single-nucleotide polymorphisms (SNPs) or small insertions/deletions that were associated with differences in human virulence in a closely related set of clinical isolates within the ST93 genotype [43]. The selective pressures applied to the *C. neoformans* cells in the host are likely very different from the pressures encountered in the environment. In the host, the yeast cell experiences high temperatures, increased CO_2_, nutrient scarcity, and attacks by the immune system. These various stressors likely drive *C. neoformans*’s adaptation for survival in the host—resulting in beneficial mutations and variations in the fungal genome. Supporting this, an additional study comparing the transcriptomes of two distantly related clinical isolates from different VN groups—one from VNI and the other from VNII—showed that stress response genes were upregulated in the patient in both isolates, but that the transporters were differently regulated [72].

*C. neoformans* also produces a wide range of phenotypes within the host. Some of these phenotypic changes include increased capsule size, melanin synthesis, and heterogeneous cell sizes, ranging from small “micro” cells to massive “titan” cells [1]. Previous reviews have covered the link between *C. neoformans* phenotype and virulence in depth [1,73]. However, a recent study investigated phenotypic variation in a collection of 70 clinical isolates and found significant correlations between cell size (either large or small), shed capsule, and poor patient outcomes [74]. In a follow-up study, the authors established an association between some of these phenotypic changes and genetic changes. By comparing the clinical isolates to the H99 derivative strains, the authors were able to show that deletion in the *SGF29* gene resulted in enhanced production of secreted capsules, increases in pleomorphic cells, and hypervirulence when compared to H99 derivatives without the *SGF29* gene deletion [75]. Similarly, loss-of-function mutations in *SGF29* were correlated with patient mortality in the clinical isolates [75].

Another intriguing factor to consider is titan cell division and poly- and aneuploidy. Titan cells are large polypoid *C. neoformans* cells that are generated during pulmonary infection and range in size from 10 to 100 µm in diameter [76,77]. While primarily characterized in murine models, titan cells are also observed in the context of human infection [78,79,80]. Titan cells are hypothesized to impact phagocytosis [81], immune responses [82], and drug resistance [83]. A key hallmark of titan cells is their polyploidy, with genomes that range from 4 °C to 312 °C [83], and the ability to produce typically sized daughter cells with reduced ploidy [76,77]. A previous study showed that titan daughter cells typically produce haploid daughter cells, but aneuploid cells that had increased drug resistance were also observed in vitro in the presence of fluconazole stress [83]. The full implications of titan cell formation the in context of infection remain poorly understood; it is not known how much genetic variation is the result of titan cells, versus the already discussed means of genetic adaptation within the host. Several studies have identified differences in titan cell formation across clinical isolates [30,84,85]. Dambuza and colleagues showed that clinical isolates with differing titan cell formation impacted disease outcomes differently in a murine model [85]. However, Mukaremera and colleagues found no correlation between in vitro titan cell formation in clinical isolates and virulence in a murine inhalation model of *Cryptococcus* [51]. These conflicting data suggest that additional studies with diverse clinical isolates are necessary to determine what role titan cells play in the pathogenesis of *C. neoformans*.

Overall, the study of clinical isolates has revealed that aneuploidy—possibly arising from titan cell formation—is hypothesized to promote adaptation and survival within the host. The beneficial gene amplifications targeted by these aneuploidies have begun to be identified in the context of fluconazole treatment and resistance. The specific genomic locations targeted by other aneuploidies observed in clinical isolates remain unknown but will undoubtedly provide invaluable information about genes that enhance virulence.

## 4. Genomic Plasticity in Clinical Isolates and Its Impact on Virulence

Analysis of *C. neoformans* environmental and clinical isolates has revealed a highly plastic genome. Comparison of serial isolates from murine models and from humans also shows evidence of chromosomal rearrangements, structural variations, and ploidy changes, with some of these genomic changes associated with disease outcomes [86,87,88]. For example, disomy of chromosome 1 is seen in reference strains exposed to the azole drug fluconazole, with this disomy thought to be driven by the presence of the *ERG11* and *AFR1* genes on chromosome 1 [87,88]. In the clinical setting, heteroresistance to azoles—defined as a subpopulation of cells that exhibit a higher level of drug resistance—is common in *C. neoformans*. Stone and colleagues showed that 12 out of 20 clinical isolates exhibited heteroresistance, and that this heteroresistance was associated with disomy of chromosome 1 [88]. Importantly, this heteroresistance is clinically relevant and influenced by drug treatment; patients treated with fluconazole alone had a higher proportion of fluconazole-heteroresistant cells compared to patients treated with fluconazole and flucytosine combination therapy [88]. While the exact mechanism underlying clinical heteroresistance and aneuploidy is unclear, deletion of the apoptosis-inducing factor gene *AIF1* in the H99 reference strain leads to production of a population of stable aneuploids that are resistant to fluconazole [89].

Aneuploidy of other chromosomes is also frequently observed. A recent study by Sephton-Clark and colleagues characterized aneuploidy in 213 clinical isolates and identified both fully and partially duplicated chromosomes in 8.5% of the isolates [90]. In this study, chromosomes 1, 9, 12, and 14 were the most frequently duplicated. Surprisingly, the growth of the aneuploid isolates was lower in vitro, suggesting that the observed aneuploidies were specifically beneficial for survival in the host [90]. In another study, Stone and colleagues observed partial and whole-chromosome copy number variations in chromosomes 1, 2, 6, 10, 11, 12, and 14; Hu and colleagues identified clinical isolates with aneuploidies and copy number variations in chromosomes 3, 4, 5, 12, 13, and 14 [28,91]. While chromosomal aneuploidies are consistently found in *C. neoformans* clinical isolates, their impact on virulence still remains unclear. For example, chromosome 13 disomy was found to be associated with reduced production of melanin and decreased virulence in mice [91], but chromosome 1 disomy was found to be associated with increased drug resistance [87,88,89].

Longitudinal studies have shown that *C. neoformans* has the ability to gain mutations over time in the host, especially in the presence of antifungal drugs. In one study, whole-genome sequencing of two isolates collected 77 days apart from the same patient revealed copy number variation on both arms of chromosome 12, as well as a protein truncation [92]. Another study analyzed 17 relapse isolates collected from patients between 55 and 409 days after the initial infection. Aneuploidy events were identified in 7 of the relapse pairs, with *ERG11* gene copy number changes observed in all 7 cases [93]. Interestingly, one relapse pair exhibited excessive ploidy changes and an increase in single-nucleotide polymorphisms and was shown to have a mutation in the mismatch repair gene *MSH2*, known to cause mutation phenotypes in other yeast species [93,94].

Overall, the study of clinical isolates has revealed aneuploidy that is hypothesized to promote adaptation and survival within the host. The beneficial gene amplifications targeted by these aneuploidies have started to be identified in the context of fluconazole treatment and resistance. However, the specific genomic locations targeted by other aneuploidies observed in clinical isolates remain unknown and will undoubtedly provide invaluable information about genes that enhance virulence.

## 5. Genetic Polymorphisms in Clinical Isolates and Their Impact on Disease Outcomes

*C. neoformans* clinical isolates are highly diverse and cause variable disease, both in human patients and in animal models. The association between Cryptococcus genotype and patient outcomes has been discussed in previous reviews [1,73,95]. In brief, several studies have shown a link between *C. neoformans* sequence type and patient outcomes, but the underlying polymorphisms that differ across the sequence types remain unknown [10,41,42,44,69]. Gerstein and colleagues performed whole-genome sequencing on 38 closely related Ugandan clinical isolates from the same sequence type (ST93) and identified polymorphisms within that population that impacted human clinical outcomes [43]. Specifically, they identified SNPs and insertions/deletions in 40 genes that were associated with patient disease characteristics such as survival and immune response [43]. Of these 40 genes, only 1 gene (*APP1*) had previously been shown to affect H99 virulence in mice [96]. However, Gerstein et al. showed that at least 35% of the identified genes impacted survival in a murine model of cryptococcosis when deleted in the KN99α genetic background [43]. Thus, genetic differences between clinical isolates can alter the host–pathogen interaction and, ultimately, affect disease outcomes.

Datasets, such as that generated by Gerstein et al., are useful to determine how closely related isolates produce variable outcomes in patients. Additional comparisons across broader spans of genetic diversity are also informative [1]. For example, a similar whole-genome sequencing study of ST5 clinical isolates compared to non-ST5 isolates from Vietnam identified translation truncation events associated with 19 genes in ST5 and 25 genes in non-ST5 isolates [97]. Interestingly, six of the genes identified by the Day study were either the same gene as that identified by Gerstein et al. or within the same intergenic region of the genome. These results suggest that specific genes or genomic regions are likely involved in adaption during *C. neoformans* infection, irrespective of genetic background. ST5 and ST93 are distantly related clades, and so the implications of the same genome regions showing alterations during disease are fascinating. Future work in diverse clinical isolates is needed to identify the frequency of adaptations in similar genes across disparate genetic backgrounds. Overall, these studies have the potential to identify the specific ways in which *C. neoformans* evolves to cause disease and, ultimately, will reveal new targets for drug development.

## 6. Use of Clinical Isolates to Study Host–Pathogen Interactions

We would like to emphasize that *C. neoformans* has a significant latency or dormancy period in humans [36,38]. *C. neoformans* seropositivity is observed in healthy children as young as 2 years old [98,99], suggesting that we encounter *C. neoformans* at a very early age. Importantly, *C. neoformans* does not elicit the same immune response between immunocompetent and immunocompromised individuals [100,101,102]. In immunocompetent individuals, the initial pulmonary infection is controlled by the host immune response through granuloma formation [15,101,103], after which it remains latent with no clinical symptoms [36]. However, individuals with primary immunodeficiencies [104,105,106,107,108,109,110], HIV-induced CD4 T-cell loss [111,112], transplant-induced immunosuppression [113], or immunodeficiency due to cancer chemotherapy [114] are missing critical immune components that control *C. neoformans*, resulting in systemic dissemination and cryptococcal meningitis. Therefore, the immune status of the host can drastically alter the disease progression and severity of *C. neoformans* infection. While advances have been made in the treatment of cryptococcal meningitis, we have yet to fully characterize the complexity of the host immune response to *C. neoformans* infection and its pathogenesis. New experimental models need to be developed that more accurately recapitulate human infection and allow us to study the nuances of clinical infection [95].

The use of reference strains to study the host immune response to *C. neoformans* infection has revealed many key insights; however, translating these findings into clinical practice has resulted in mixed success. For instance, mice infected with a genetically engineered *C. neoformans* H99 reference strain expressing the type-1 cytokine IFNγ are able to clear the infection, suggesting that IFNγ supplementation may have therapeutic benefits for treating cryptococcal meningitis [115]. However, in a clinical trial, adjunctive IFNγ treatment did not significantly improve patient survival, even though the rate of fungal clearance was faster compared to the standard treatment control group [116]. While IFNγ is important in host defense against *C. neoformans* infection, clinical data indicate that questions remain as to the role of IFNγ in each stage of *C. neoformans* disease progression—from latent infection to disseminated cryptococcal meningitis. These challenges also apply to other immune components that have been identified as important in the defense against *C. neoformans* [117,118,119,120,121]. In most studies using reference strains, a lethal infection model is used in which immunocompetent mice are infected with the H99/KN99α reference strain and succumb to cryptococcal meningitis. It is important to note that the host response to lethal infection in an immunocompetent murine model may be quite different from the immune response in an immunocompromised human or animal model. In contrast to healthy humans, immunocompetent mice infected by the commonly used reference strains H99/KN99α ultimately succumb to the infection irrespective of inoculum level and, thus, are thought to represent the immune responses typically observed in immunocompromised individuals [37].

Historically, other mammalian models, such as guinea pigs, rabbits, and rats, have been used to study the host immune components that are critical for controlling *C. neoformans* infection [122]. While the immune responses in these immunocompetent mammalian models are more consistent with the controlled infections observed in healthy humans, these models are limited by a lack of advanced immunological and genetic tools [122]. Furthermore, while the reference strain *C. deneoformans* 52D can establish latent infections in rats and mice [38,39,40], this strain undergoes genetic and phenotypic changes affecting its virulence under standard laboratory conditions [123], and *C. deneoformans* only accounts for 4% of all infections caused by the *Cryptococcus* species complex [124]. Thus, while the use of reference strains in animal models of cryptococcosis offers consistency between studies, we should keep in mind the caveats of using reference strains to study the host immune response to *C. neoformans* infection from both the host and pathogen perspectives.

**Figure 2:**
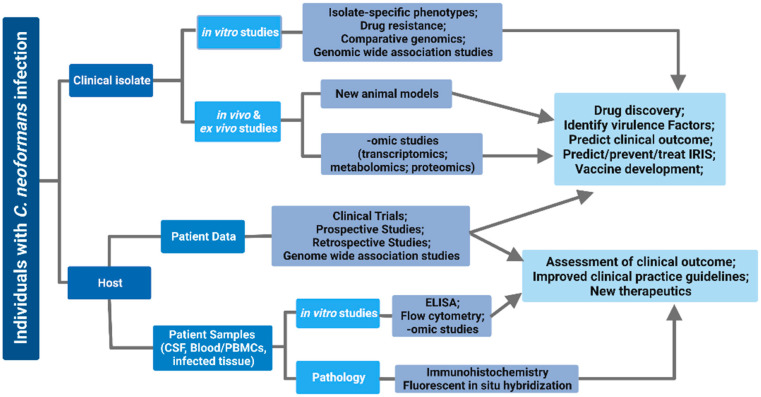
Experimental strategy to study host–pathogen interactions using *C. neoformans* clinical isolates.

There are several advantages to using clinical isolates in studies of the host immune response to *C. neoformans* infections, including identification of strain-specific differences, clinical relevance, and the development of new experimental models (Figure 1 and Figure 2). Human patients infected with *C. neoformans* strains within the same sequence type frequently do not have similar clinical outcomes [42,43,51], suggesting that strain-specific differences in *C. neoformans* clinical isolates may impact disease outcomes. Studying the differences in virulence and pathogenesis between clinical isolates may allow us to identify host and pathogen factors that are beneficial or detrimental during human infection. Indeed, studies have already begun to use clinical isolates to identify factors that influence *C. neoformans*’s virulence. The observation that clinical isolates had different rates of non-lytic exocytosis from macrophages led to the identification of melanization and laccase activity as drivers of *C. neoformans*’s virulence [125]. Clinical isolates also show variable phagocytic uptake and intracellular proliferation within macrophages, and isolates that have low phagocytic uptake and intracellular proliferation are associated with non-sterilization of cerebral spinal fluid after antifungal therapy in patients [126,127]. In a separate study, clinical isolates that displayed increased phagocytic uptake by macrophages were found to induce M2 macrophage polarization, a type-2 immune response, and poor survival outcomes in mice [128]. Other studies have also found that clinical isolates that cause early mortality in mice are resistant to reactive nitrogen and oxygen intermediates [129]. Altogether, these findings suggest that clinical isolates derived from patients with poor clinical outcomes can better evade macrophage phagocytosis, resist reactive nitrogen and oxygen production, and promote M2 macrophage polarization. Future work is needed to understand how *C. neoformans* modulates these aspects of the immune response during infection, and to identify the genetic differences in clinical isolates that affect the immune response and, ultimately, the clinical outcome.

The murine inhalation model shows a strong association between human and murine mortality rates with *C. neoformans* clinical isolates, suggesting that this model is able to recapitulate human disease [51], and it was used to identify clinical isolates that produce latent infections in mice, broadening our ability to study latent infections and model the transition to lethal disease in immunocompromised hosts [35]. Importantly, the latent clinical isolates establish a persistent pulmonary infection that is controlled within granulomas in immunocompetent mice, mirroring many aspects of infection in healthy humans [35]. Upon immune depletion of these latently infected mice, the infection progresses to disseminated lethal disease similar to that observed in immunocompromised humans. Further identification and analysis of the continuum of disease observed with *C. neoformans* clinical isolates will create a large repository that can be used to identify isolate characteristics that promote persistence within the host [95]. In fact, initial studies utilizing clinical isolates have already identified ex vivo capsule size [130], laccase activity [127], cytokine responses [41], and regulatory T-cell activity [131] as factors affecting clinical outcomes in immunosuppressed patients with cryptococcosis. In addition, recent GWAS studies comparing genetic differences between clinical isolates have started to identify possible underlying genetic differences between the isolates that likely produce the observed differences in patient clinical outcomes [43,90].

The question of which clinical isolates to use in specific experiments is worth considering. Though under-studied, characterizing the phenotypic and genetic differences between isolates collected from different infection sites is a potentially fascinating avenue of research [132]. For example, while phenotypic differences between isolates from different organs have been identified, the underlying genetic differences and/or whether these observations are consistent across clinical isolates is unknown [133]. It is well reported that sequence type matters in the context of disease [10,41,42], especially when considering the ST5 isolates that cause primary infection in Vietnam [44], but these studies are still in their infancy. While the exact clinical isolates to be used will likely vary based on the scientific question to be addressed, the isolates should possess the following characteristics: (1) whole-genome sequencing; (2) metadata that include human clinical data and outcomes; and (3) a core set of in vitro, in vivo, and in silico analyses that can be used for cross-study comparisons. Ideally, these data will be fully annotated and easily accessible for each clinical isolate in existing databases such as FungiDB [134] or the Multi-Locus Sequence Typing database [135]. Overall, studies with clinical isolates will be crucial to the identification of pathogenic and host factors that influence the severity of *C. neoformans* infection, as well as being instrumental in determining the clinical outcomes of patients and in the administration of appropriate therapies to improve patient survival (Figure 2).

## 7. Conclusions

In summary, the study of reference strains has expanded our understanding of *C. neoformans* infections and provided a basic understanding of the key virulence factors and traits that are critical for disease. In addition, functional studies with reference strains will continue to play a critical role in understanding the role of specific genes in *C. neoformans*’s biology and pathogenesis. However, expanding our studies to include comparative analyses of clinical isolates will greatly expand our knowledge and will identify critical components of the host–pathogen interactions that have been overlooked to date.

## Data Availability

Not applicable.
